# Introducing fairness in Norwegian air ambulance base location planning

**DOI:** 10.1186/s13049-021-00842-0

**Published:** 2021-03-20

**Authors:** Caroline J. Jagtenberg, Maaike A. J. Vollebergh, Oddvar Uleberg, Jo Røislien

**Affiliations:** 1 School of Business and Economics, Vrije Universiteit Amsterdam, Amsterdam, The Netherlands; 2 Faculty of Engineering, University of Auckland, Auckland, New Zealand; 3 Faculty of Electrical Engineering, Mathematics & Computer Science, Delft University of Technology, Delft, The Netherlands; 4 Department of Research, The Norwegian Air Ambulance Foundation, Bergen, Norway; 5 Department of Emergency Medicine and Pre-Hospital Services, St. Olav’s University Hospital, Trondheim, Norway; 6 Faculty of Health Sciences, University of Stavanger, Stavanger, Norway

**Keywords:** HEMS, Air ambulance, Facility location problem, Fairness

## Abstract

**Background:**

A primary task of the Norwegian helicopter emergency medical services (HEMS) is to provide advanced medical care to the critical ill and injured outside of hospitals. Where HEMS bases are located, directly influences who in the population can be reached within a given response time threshold and who cannot. When studying the locations of bases, the focus is often on efficiency, that is, maximizing the total number of people that can be reached within a given set time. This approach is known to benefit people living in densely populated areas, such as cities, over people living in remote areas. The most efficient solution is thus typically not necessarily a fair one. This study aims to incorporate fairness in finding optimal air ambulance base locations.

**Methods:**

We solve multiple advanced mathematical optimization models to determine optimal helicopter base locations, with different optimization criteria related to the level of aversion to inequality, including the utilitarian, Bernoulli-Nash and iso-elastic social welfare functions. This is the first study to use the latter social welfare function for HEMS.

**Results:**

Focusing on efficiency, a utilitarian objective function focuses on covering the larger cities in Norway, leaving parts of Norway largely uncovered. Including fairness by rather using an iso-elastic social welfare function in the optimization avoids leaving whole areas uncovered and in particular increases service levels in the north of Norway.

**Conclusions:**

Including fairness in determining optimal HEMS base locations has great impact on population coverage, in particular when the number of base locations is not enough to give full coverage of the country. As results differ depending on the mathematical objective, the work shows the importance of not only looking for optimal solutions, but also raising the essential question of ‘optimal with respect to what’.

**Supplementary Information:**

The online version contains supplementary material available at 10.1186/s13049-021-00842-0.

## Background

Emergency medical services (EMS) are an important and integrated part of health services in most countries [1]. As a supplement to EMS, helicopter emergency medical services (HEMS) are expanding throughout the world, particularly in high-income countries [2, 3]. The main purpose of HEMS is to provide advanced point-of-care diagnostic modalities, complex clinical decision-making, advanced interventions beyond the scope of most EMS, shorter transport times and access to locations outside the roadmap [4, 5]. The service is resource-intensive and limited [2] so in order to optimize its utilization the location of HEMS bases is crucial.

In Norway HEMS is considered essential in order to ensure equal access to specialized healthcare throughout the country [6]. HEMS performance is typically measured in terms of response times, that is, the time from emergency call to helicopter on-scene arrival. A 2001 government white paper states that 90% of the population should be reached by a physician-manned ambulance within 45 min [7]. Such performance targets have led to the search for *efficient* configurations. That is, how the target can be met using a minimal number of bases and vehicles, or how to maximize coverage with a given number of bases and vehicles. This focus on efficient use of HEMS in Norway has been applied in several studies challenging the current locations of HEMS bases in Norway, either in greenfield scenarios or by making small adjustments to the current system [5, 8]. Optimizing for efficiency has also been seen in studies of ambulance bases in Canada [9] and Italy [10] as well as fire fighter bases in the Netherlands [11]. In a review of ambulance optimization methods all 17 methods mentioned optimized for efficiency [12].

While maximizing efficiency seems reasonable, such mathematical models will inadvertently put more focus on people in densely populated areas. By deciding to strive for efficiency one therefore implicitly affects who gets coverage and who does not [13]. Indeed, a less efficient solution might be preferable if it offers a more equitable service. EMS providers around the world recognize fairness as a relevant factor [13] and in Norway in particular, where despite substantial differences in geography and population density equality in healthcare is a spoken aim.

To include fairness in EMS systems design, recent literature looked at welfare economics [14]. This field deals with how to combine individual preferences to make a joint decision, with individual satisfaction with the ambulance system combined in a so-called *social welfare function* (SWF). Several social welfare functions exist, each representing different ways of making joint decisions. Efficiency and fairness are entailed in this framework, corresponding to two different SWFs.

The aim of this paper is to present an alternative to the practice of optimizing base locations for efficiency only, and demonstrate how the concept of fairness can be included in the corresponding mathematical models. We compute and compare optimal HEMS base locations for Norway using three different SWFs, which represent different levels of aversion to inequality.

## Methods

### Setting

**Fig. 1:**
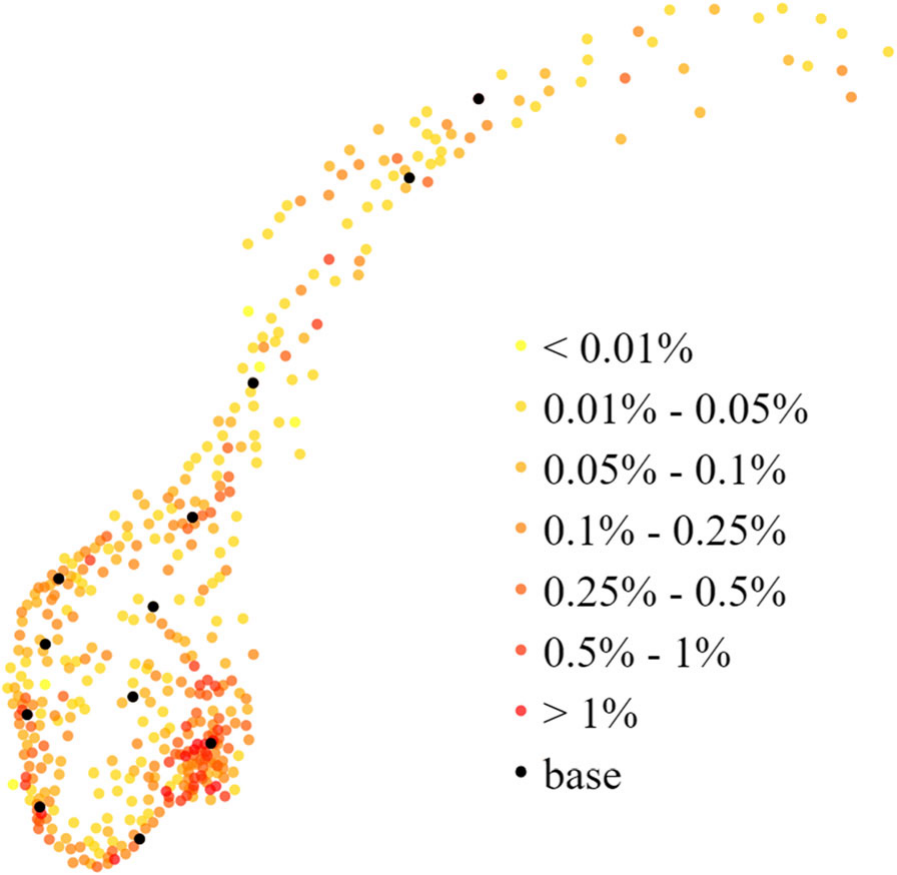
Population density heat map of Norway. Each municipality is depicted as a dot located in the population-weighted centroid of the municipality. The color indicates the fraction of the population living in that municipality in regards to the total Norwegian population. The twelve existing air ambulance bases are superimposed

Mainland Norway stretches 1790 km from north to south, covering 323,780 km^2^ at the far North of Europe. The population was 5.2 million on January 1st 2015 [15], with county population density ranging from 1129.5 inhabitants/km^2^ in Oslo to 1.5 inhabitants/km^2^ in the northernmost county Finnmark. For mathematical modelling of HEMS base locations in Norway, the difference between using municipality data and fine grid data is negligible [8] and the present study used municipality level population data as this reduces computation times. Data are freely available from Statistics Norway [15]. For our study we used the 428 municipalities that constituted Norway in 2015, each represented as a population-weighted centroid (the population centre). Municipality population density of Norway is shown in Fig. 1.

Norway has a publicly funded national anesthesiologist-manned air ambulance network consisting of 12 HEMS bases [16]. In addition, the country has seven fixed wing bases and six search and rescue helicopter bases (both out of scope for our study). The objective of the National air ambulance service is to provide advanced medical care to critically injured or ill patients. The service operates 24/7/365.

### Response times

A response time is defined as the time from an emergency call to the moment of helicopter arrival on scene. It consists of a reaction time – which includes the essential preparations for a flight - followed by a flight time.

To model the expected response time from any potential base to any municipality, we first look at reaction times. We used an average of 5.5 min found in an empirical study [6]. Next, we estimated flight times by computing the distance between two locations and dividing this by an average helicopter speed of 220 km/h, a number also found in other Norwegian HEMS studies [5].

The method above gives reasonable estimates for *average* response times, however, in reality response times are stochastic in nature. Reaction times depend on readiness of crew and helicopter, and flight times are affected by various factors such as wind direction and strength [8]. There is no literature on how helicopter flight times are distributed in Norway. Several studies on other HEMS systems have reported considerable variation in response times [17, 18], but remain undetermined as of how much of the variation is due to the variation in reaction time or flight time. In a Swedish study [19] flight times appear evenly distributed in a band around the linear regression line, suggesting a uniform distribution. In the present calculations, we primarily modelled response times as uniformly distributed with at most a 10% deviation from the expected value. Other similar models were also explored, see Supplementary file ‘additional computations’.

### Utility

We address fairness for HEMS in Norway by applying the ideas in [14]. This includes the key idea to view the system from a perspective of welfare economics, which deals with how to combine individual preferences to make a joint decision. In this framework, each individual has their preferences expressed as a numerical value called a *utility* representing their satisfaction with the ambulance system.

The main performance indicator for Norwegian HEMS is whether or not response times are within 45 min [7]. This, combined with stochastic flight times, leads us to define an individual’s utility as the probability that a helicopter departing from the closest base arrives within 45 min.

These utilities are input for a so-called *social welfare function* (SWF), which prescribes how to combine the individual utilities and returns as output a numeric representation of the collective welfare of the group [20, 21].

### Mathematical modelling

A general facility location model optimizes the locations of a fixed number of bases with respect to some objective mathematical criterion [22]. Many such models implicitly assume that whenever a patient needs HEMS, a helicopter is available at the closest base. In this sense such models represent a best case scenario [8]. Facility location models can work with a range of possible objectives, depending on how one wants to weigh efficiency versus equity among the population. In the context of collective decision making, each of those objectives is a SWF [20]. It has been argued that SWFs should be used as a framework for governmental policy analysis in particular [23]. In the current work we explore three different SWFs, namely one for efficiency, one for fairness, and one that balances efficiency and fairness. This allows us to determine optimal helicopter base locations, but optimized with respect to different levels of aversion to inequality.

#### Utilitarian

Base location models commonly optimize on efficiency, which corresponds to using a utilitarian SWF [14]. The utilitarian SWF defines the welfare of the group as the average of all individual utilities. Focusing on the average achieves “the greatest happiness for the greatest number of people”, however, note that the average behaves as follows. Increasing the utility of one individual contributes the same to the average regardless of whether that individual is already well off compared to the rest of the population or not. When phrased in terms of wealth: whether the rich become richer, or the poor become richer, in a utilitarian world both options are equally good. Maximizing a utilitarian SWF is generally described as optimizing on efficiency, or maximizing total coverage. The approach maximizes the number of demand locations covered, weighted by the demand in each demand location, and typically leads to bases in areas with high population densities.

#### Bernoulli-Nash

The Bernoulli-Nash SWF puts more weight on individuals with low utility values than does the utilitarian SWF. The Bernoulli-Nash SWF can be thought of as a ‘no man left behind’ approach: if any individual has utility zero, the social welfare of the whole group is zero as well. Moreover, it weighs options when presented a choice between a large increase in utility for an individual who is already well off, or a small increase in utility for an individual at the lower end of the spectrum. Optimizing the Bernoulli-Nash SWF is defined as maximizing the product of the utilities. The approach was recently proposed for the problem of ambulance location, where it was demonstrated that the Bernoulli-Nash SWF corresponds to a mathematical formulation of the concept of fairness [14]. If every individual makes exactly one ambulance call, the Bernoulli-Nash SWF is the joint probably that *everyone* receives their ambulance on time. Compared to a utilitarian approach, the Bernoulli-Nash SWF is averse to inequalities, and the Bernoulli-Nash optimum places more ambulances in areas with lower population density. Even people who are hard to reach deserve some coverage.

The Bernoulli-Nash SWF has one disadvantage in being sensitive to zeroes [14]. Since one demand point with zero utility results in an overall objective value of zero, the Bernoulli-Nash SWF is unable to distinguish between scenarios with one zero utility or multiple zero utilities. The Bernoulli-Nash SWF is therefore most suitable for optimizing base locations in a scenario where everyone can be reached on time with some non-zero probability. This is not the case for Norway, at least not without a drastic increase in bases, and the Bernoulli-Nash SWF is thus of limited practical value for the present analysis.

#### Iso-elastic

The utilitarian and Bernoulli-Nash SWFs can be viewed as representing two ends of a spectrum, where a utilitarian SWF is focusing on average happiness of the group, while the Bernoulli-Nash SWF is balancing the desire to have a high average happiness and simultaneously having an aversion to inequity between individuals.

The iso-elastic SWF is a flexible SWF that creates a continuum between the utilitarian and Bernoulli-Nash SWFs [24]. See Appendix 1 for a formulation and an illustrative example. This SWF includes a parameter *a*, taking values from 0 through 1, quantifying the decisionmaker’s aversion to inequality. For *a = 0* the iso-elastic SWF is equal to the utilitarian SWF, and in the limit where *a* approaches 1 it becomes the Bernoulli-Nash SWF, see Supplemental file “proof of convergence”. Choosing *a* close to 1 yields a SWF that preserves most of the fairness properties of Bernoulli-Nash, but without the sensitivity to zeroes.

### Computations

All 428 municipalities in Norway are used as both demand locations and potential base locations, where the demand in each municipality is modelled as the fraction of the Norwegian population living there. The mathematical optimization model and the response time model are combined to determine optimal base locations according to the different SWFs.

First we performed a greenfield analysis, computing optimal base locations as if no current bases exist. Moreover, we performed conditional optimization, exploring what the optimal relocation or addition of one or two bases would be given the existing twelve bases in Norway. We computed results using the existing response time target of 45 min. There is however also significant interest in exploring the practical consequences of lowering the response time threshold to 30 min [25], so we used that target time in additional computations.

The models were implemented in Julia [26] and solved with Gurobi [27]. Since the Bernoulli-Nash and iso-elastic objective functions are non-linear, those optimization models are not solvable by standard off-the-shelf solvers. To overcome this, we approximated the objective function by a piecewise linear function, an approach also used previously [14], which allowed Gurobi to solve the models. For technical details, see Supplementary file.

## Results

Results for the different scenarios are described below.

### Optimization in greenfield scenarios

**Fig. 2:**
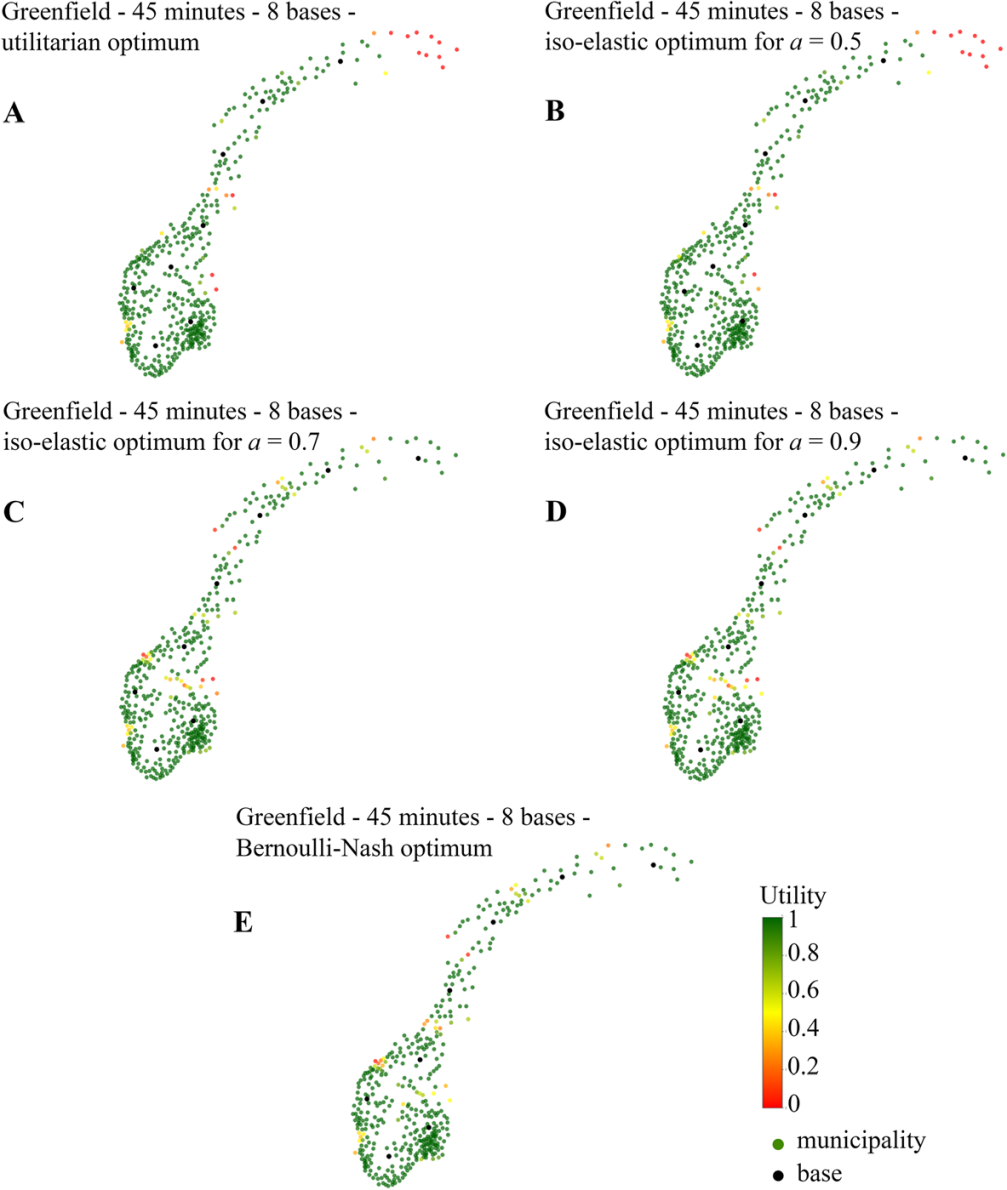
The optimal 8 base locations for a time threshold of 45 min using different objective functions. Utility is defined as the probability that a HEMS departing from the nearest base reaches the patient on time; green means an inhabitant may expect HEMS to always be on time; yellow means inhabitants should expect on-time arrival in 50% of the cases, and red 0% of cases

The greenfield analysis for a time threshold of 45 min was done with eight bases, as previous work [5] has shown that eight bases is enough to cover 95% of the population in 45 min. The optimal locations of air ambulance bases using the traditional utilitarian SWF are shown in Fig. 2a. While large parts of the country are fully covered on time (depicted in green), note that the most northern part of Norway is left uncovered, that is, the probability of reaching patients there on time is zero. This is the case for 13 of the 428 municipalities (depicted in red). Compare this to the Bernoulli-Nash optimum in Fig. 2e in which we observe all municipalities have a non-zero probability of being reached on time.

Optimal solutions for iso-elastic SWFs with *a* = 0.5, 0.7 and 0.9 are shown in Fig. 2b, c and d, respectively. As *a* is getting closer to 1, increasingly more weight is put up north. Results for *a* = 0.5 are very similar to the utilitarian optimum, the only difference can be found the south, where two base locations are shifted to provide some coverage to a previously uncovered municipality. For *a* = 0.5 there were 12 uncovered municipalities, while there is only one uncovered municipality for *a* = 0.7. Results for *a* = 0.9 are similar to *a* = 0.7 and change only slightly when using the Bernoulli-Nash objective function.

### SWF comparisons in greenfield scenarios

**Fig. 3:**
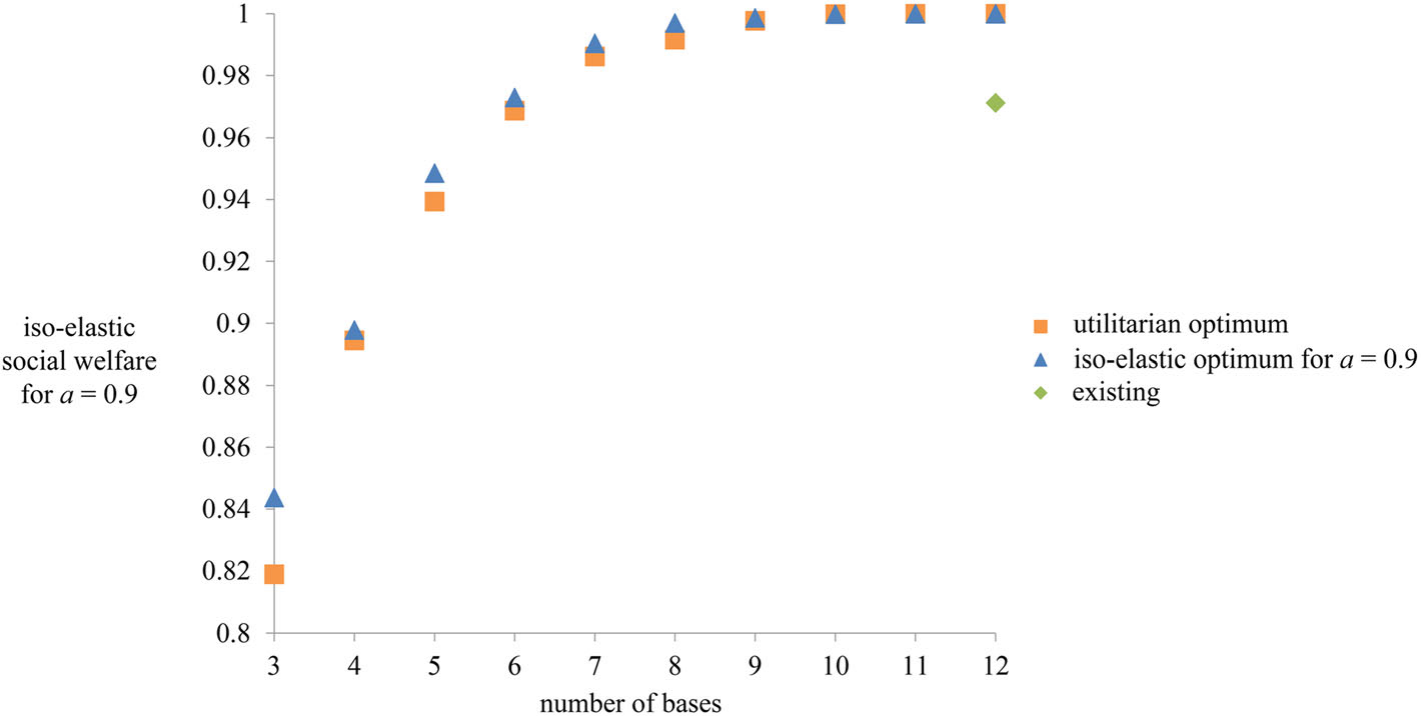
Iso-elastic social welfare that is achieved by the utilitarian and iso-elasticoptimum for *a* = 0.9, for increasing number of base locations

The utilitarian and the iso-elastic SWF give different optimal solutions for HEMS base locations. We compare solutions for the utilitarian and iso-elastic SFW with *a* = 0.9 and a time threshold of 45 min, while varying numbers of bases from 3 to 12 – the current number of HEMS bases (Fig. 3). The gap between the utilitarian and the iso-elastic optimum is bigger when the number of bases is small. Also observe that the performance of the existing base structure is sub-optimal in the sense that the iso-elastic social welfare achieved by the twelve current bases can already be achieved by just six differently positioned bases.

### Optimization conditioned on existing base structure

**Fig. 4:**
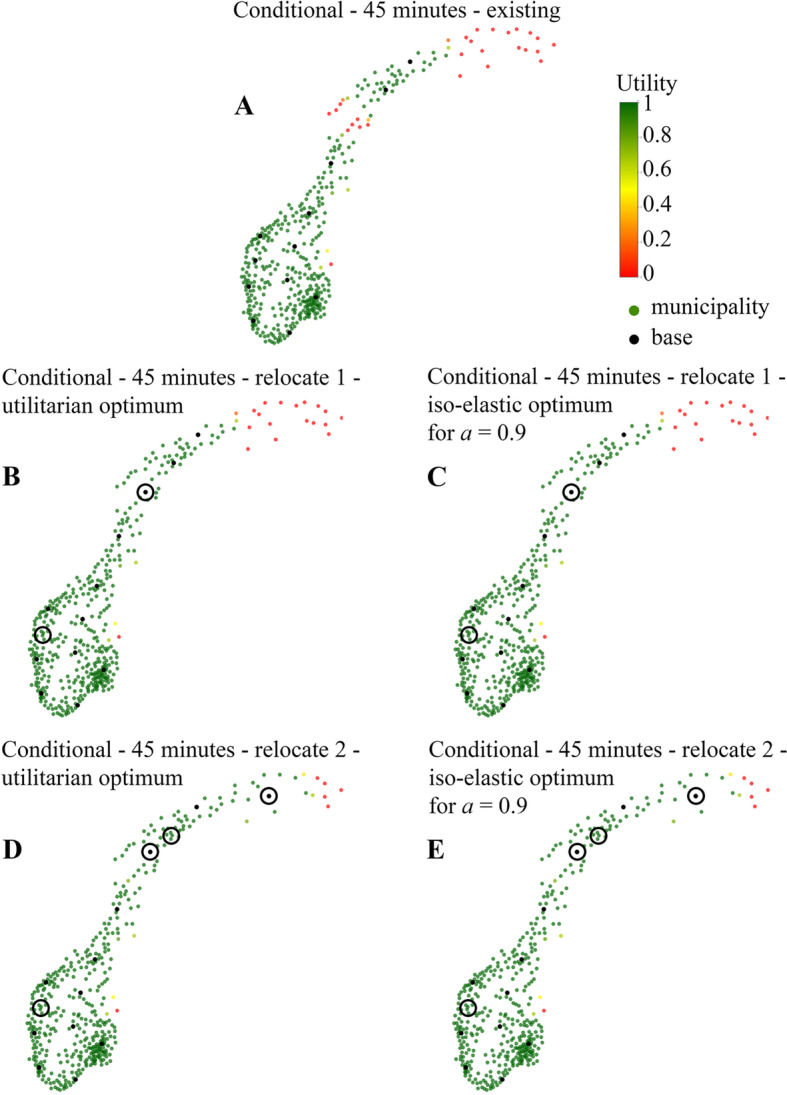
Optimal base locations when relocating one or two bases compared to the existing base structure for a 45 min threshold, using different objective functions. Utility is defined as the probability that a HEMS departing from the nearest base reaches the patient on time; green means an inhabitant may expect HEMS to always be on time; yellow means inhabitants should expect on-time arrival in 50% of the cases, and red 0% of cases

First, we examine the existing base structure under a 45 min threshold. It fully covers the south, except for two municipalities northeast of Oslo (Fig. 4a). One of the bases in western Norway is redundant, and northern Norway shows two regions without coverage.

Next, we analyse the optimal relocation of one base. We calculate this for both the iso-elastic SWF with *a* = 0.9 and the utilitarian SWF, which turn out to give the same solution (Fig. 4b and c), with the redundant base along the west coast relocated to one of the uncovered regions in northern Norway. Relocating two bases also gives the same result in both the utilitarian and the iso-elastic case (Fig. 4e and f) with almost all municipalities covered.

**Fig. 5:**
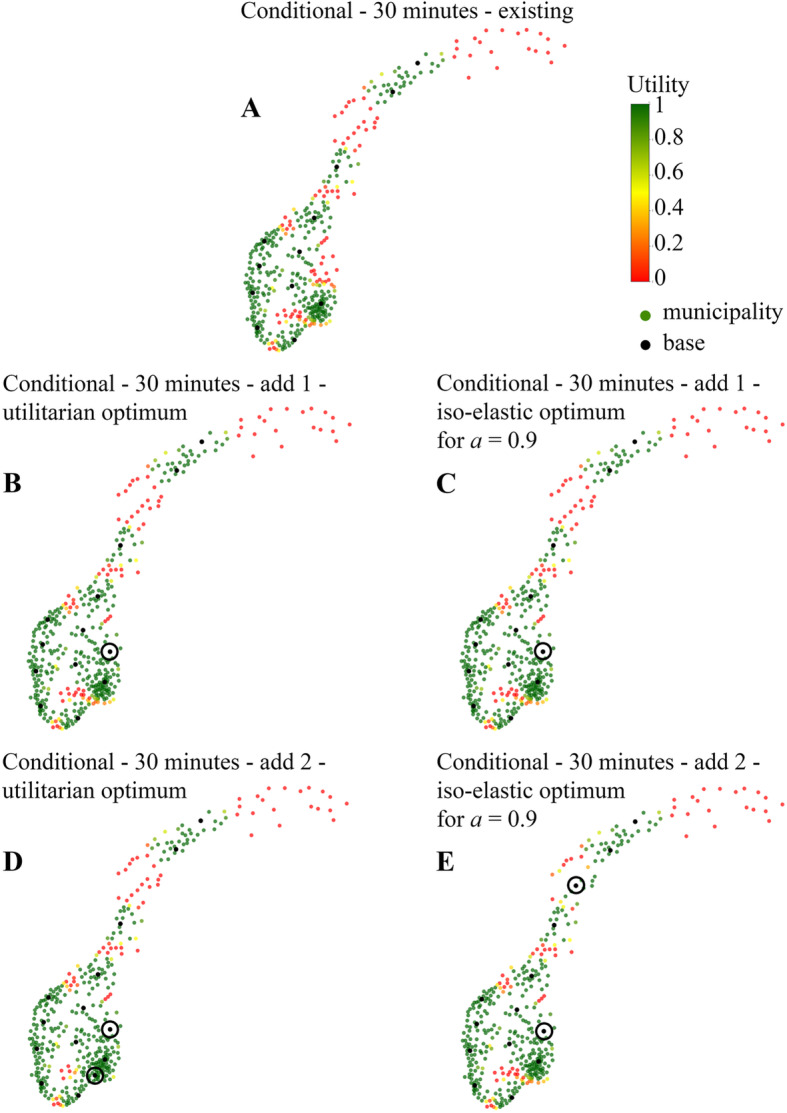
Optimal base locations when adding one or two bases to the existing base structure for a 30 min threshold, using different objective functions. Utility is defined as the probability that a HEMS departing from the nearest base reaches the patient on time; green means an inhabitant may expect HEMS to always be on time; yellow means inhabitants should expect on-time arrival in 50% of the cases, and red 0% of cases

Lowering the threshold to 30 min, the existing 12 bases cover fewer municipalities and the uncovered municipalities are more spread around the country (Fig. 5a). We next investigate the addition of bases. Adding one base gives the same result for both the utilitarian and the iso-elastic case, with the extra base positioned north of Oslo (Fig. 5b and c). Adding two bases, one is placed in the same location for both cases, while the second base depends on the objective function. The utilitarian model places the second base in the Oslo region, while the iso-elastic model places it in the northern part of the country (Fig. 5d and e).

### Response time distributions

In the above analysis we modelled response times as uniformly distributed with up to 10% deviation from the expected value. Experimenting with different response time distributions we found that having more variation in response times results in more bases in areas with high population densities (not shown). This is true both for the utilitarian and the iso-elastic welfare. This implies that a realistic response time distribution is important for determining the exact position of bases. The general tendency, that an iso-elastic optimum puts more bases in low population density areas than a utilitarian optimum would, does however remain.

### Solve times

Solve times depended on the objective function and type of analysis performed. Utilitarian models are the fastest and generally solve within seconds. The longest solve times were observed for iso-elastic models where the parameter *a* is close to 1. For example, a greenfield analysis for *a* = 0.9 (8 bases, time threshold 45 min) took 6.7 min to solve, while the utilitarian counterpart took only 14 s. These timing results were obtained on a MacBook pro 2.6 ghz 6-core intel core i7 with 16 GB memory. All of our computations were much faster than the 2–5 h reported in [14], because in that work the authors also computed the optimal number of vehicles per base.

## Discussion

When maximizing fairness for Norwegian HEMS rather than efficiency we find that bases are more spread around the country, and avoid the typical clustering of bases around urban areas (Fig. 2).

For optimal relocation of one or two bases under a 45 min time threshold, solutions were the same for both a utilitarian and an iso-elastic objective function (Fig. 4b-e). This should be comforting for decision-makers; there is *one* optimal decision, regardless of one’s aversion to inequality. Notably, in this case study all tested SWFs leave the far north of the country uncovered. The interpretation is that the coverage achieved further south serves so many more people than those that could possibly be helped up north, that even equity concerns cannot outweigh this benefit in coverage.

The iso-elastic model to some extent captures the ‘no man left behind’ property that we have seen in the Bernoulli-Nash model before. This can be observed in the computation where we added two bases to the current configuration, using a 30 min response time threshold. The iso-elastic optimum in Fig. 5e shows quite some municipalities with a utility around 0.5 (depicted in yellow) in the Oslo area, where the population density is high. Conversely, the utilitarian optimum in Fig. 5d shows more municipalities with a utility close to zero (depicted in red) further north, where fewer people live. This can be explained as follows. The utilitarian model noticed that adding bases in the Oslo region improves HEMS service levels in that area from moderate to great, and many people live there, hence it considers this the best decision. The iso-elastic model on the other hand, puts more emphasis on helping people who are currently experiencing very poor HEMS service levels and raise this to at least a moderate level - even if the affected group up north is smaller than the group near Oslo.

This is the first study proposing to solve facility location models using an iso-elastic SWF. Here it was a useful alternative to the Bernoulli-Nash SWF. The iso-elastic SWF depends on a fixed parameter *a*, and we experimented with several values. Choosing *a* close to 1 preserved most of the desired fairness properties of the Bernoulli-Nash SWF, while avoiding the numerical issues that would occur for Bernoulli-Nash in case studies where it is impossible to cover every municipality.

Most of our calculations were done under the assumption that response times are uniformly distributed. While the present work is able to determine general trends, the exact locations of bases in the optimal solution require further research. In particular, the questions (1) how the results of the model would change in dependence of the response time modelling, and (2) what is an accurate response time distribution for Norwegian HEMS, are important directions for future work. Subsequently, any given response time distribution would then be straightforward to combine our models.

The models presented in this paper are appropriate to determine where to locate HEMS bases, however, to analyse how many helicopters to allocate to each base these models need extending. It then becomes necessary to model the fact that helicopters are sometimes unavailable, which can be done by including a predetermined *busy fraction*. This has been demonstrated in both a model for efficiency [28] and a model for fairness [14]. Note that due to the large rural-urban differences in Norway, it is unclear whether a busy fraction model is appropriate, and modelling HEMS unavailability remains a topic for careful consideration.

We introduced fairness in a base location study for Norwegian HEMS, considering objectives with different measures of aversion to inequality. The choice of objective function can severely affect study outcomes, especially when the available number of bases is not enough to fully cover the whole country with respect to the given target time. Where utilitarian solutions display a strong focus on densely populated areas at the cost of rural areas, this is avoided when fairness is captured in the model. For Norway including fairness increases service levels in the north of the country.

Deciding on what is the correct objective function is not straightforward, and includes careful consideration of what price one is willing to pay for fairness. The answer will likely be different in different countries and political climates.

## Conclusions

This work shows how clinical and subjective opinions can be implemented in mathematical models that determine optimal ambulance base locations. It demonstrates that the choice of objective function can severely affect study outcomes, and thereby challenges us to question what we mean by the “best” or “optimal” solution. We advise to reconsider the current practice of optimizing base locations for efficiency only.

### Supplementary Information

**Additional file 1.**

**Additional file 2.**

**Additional file 3.**

## Figures and Tables

**Table 2:**
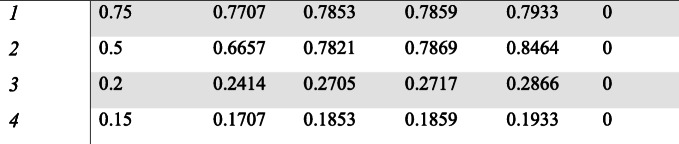
Objective values of the different SWFs for every possible base position

The maximum value per column is highlighted

**Fig. 6:**
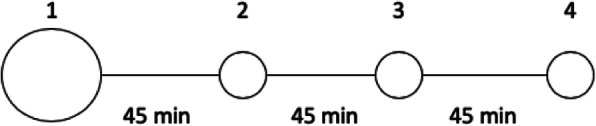
Small example for the ambulance location problem, consisting of four demand locations (municipalities) which are all potential base locations

## Data Availability

Municipality data are freely available from Statistics Norway [15], including population numbers and locations. Location data can be converted to expected HEMS response times using the calculation described in the Material and method section; however, these expected response times are also available from the corresponding author on reasonable request.

## Appendix 1

In this appendix we provide the formal definition of the so-called iso-elastic social welfare function (SWF). Moreover, we present a small example that serves to demonstrate the behaviour of the three different SWFs mentioned in this paper.

### Iso-elastic social welfare function

The iso-elastic SWF [24] combines individual preferences within a social group to make a joint decision about the satisfaction with the system under study. When placed in the context of Norwegian HEMS base locations it reads as follows.

$$ \frac{1}{1-a}\sum \limits_{\left\{i=1\right\}}^{\left\{428\right\}}{d}_i{u}_i^{1-a} $$

Where the utility *u*_*i*_ of a patient in municipality *i* is defined as the probability of a helicopter departing from the nearest base reaching *i* within the response time threshold. Moreover, *d*_*i*_ is the fraction of demand in municipality *i* and *a* is a predefined parameter between zero and one. In our numerical work – and the results reported in this paper - we removed the factor 1/1-*a* from the equation, because this makes sure outcomes of the iso-elastic SWF are between 0 and 1, hence making it easier to compare to other SWFs which operate on the same 0 to 1 scale. Note that this change does not affect where the optimal base locations are.

### Illustrative example

Consider four municipalities positioned on a line (Figure A1 in Appendix), with 70% of the demand in the first circle and 10% in each of the remaining three. Flight times are uniformly distributed with expected values as depicted in the figure. In this example the reaction time is 0. The task is to open a HEMS base in one of the four municipalities, and patients evaluate their service by the probability of being reached within 45 min. Table 1 shows for each choice the corresponding utilities for individuals in each municipality. This leads to the values attained for the group by the different SWFs in Table 2, which shows that the utilitarian optimum is to build the base at municipality 1. In contrast, the Bernoulli-Nash SWF is not able to distinguish between the different base locations, as there will always be a municipality with utility 0. The iso-elastic SWF for small *a* gives the same result as the utilitarian SWF, while for large *a* the optimum is to build the base at municipality 2.

**Table 1:** Utilities of inhabitants of each municipality for every possible base position

Position base	Municipality 1	Municipality 2	Municipality 3	Municipality 4
1	1	0.5	0	0
2	0.5	1	0.5	0
3	0	0.5	1	0.5
4	0	0	0.5	1

## Abbreviations

EMS: Emergency medical services
HEMS: Helicopter emergency medical services
SWF: Social welfare function
