# Supplementary material for: Introducing fairness in Norwegian air ambulance base location planning
Source: Scand J Trauma Resusc Emerg Med. 2021 Mar 20;29:50. doi: 10.1186/s13049-021-00842-0 (PMC7980553; doi:10.1186/s13049-021-00842-0)
Supplement: Supplementary file 1 — Additional file 1. [file 13049_2021_842_MOESM1_ESM.pdf]

Technical details, supplemental to:

C. J. Jagtenberg et al.

Introducing fairness in Norwegian air ambulance base location planning

In this file we formulate the mathematical model that maximizes the iso-elastic SWF and give details on how we solved it.

Indices:

i = demand zone: i ∈ V.
j = possible base location: j ∈ W.

For sets V and W we both used the municipalities of Norway, numbered 1 to 428.

Parameters:

N = number of base locations to be opened.
T = response time threshold.
d_i = fraction of demand in municipality i.
P_{ij} = probability that the response time is smaller or equal to T, for a patient in municipality i and a helicopter departing from base j.

Decision variables:

$$x_j = \begin{cases} 1, & \text{if } j \text{ is a base,} \\ 0, & \text{otherwise.} \end{cases}$$

$$z_{ij} = \begin{cases} 1, & \text{if the closest air ambulance for demand location } i \text{ is located at possible base location } j, \\ 0, & \text{otherwise.} \end{cases}$$

u_i = utility for a patient at demand zone i.

Objective and constraints:

Maximize $\sum_{\{i=1\}}^{\{428\}} d_i u_i^{1-a}$

Subject to:

(1) $\sum_{j \in W} x_j \le N,$

(2) $\sum_{j \in W} z_{ij} = 1,$ for i ∈ V

(3) $z_{ij} \le x_j,$ for i ∈ V, j ∈ W

(4) $u_i \le \sum_{j \in W} p_{ij} z_{ij},$ for i ∈ V

$x_j \in \{0,1\}$, for j ∈ W
$z_{ij} \in \{0,1\}$, for i ∈ V, j ∈ W
$u_i \in [0,1]$, for i ∈ V.

Here, constraint (1) prescribes that we may open no more than N bases. Constraint (2) makes sure that for each demand zone, only one base is the closest. Constraint (3) ensures that the closest base can only be found in a location that actually is assigned as a base. Finally, constraint (4) defines the utility of a patient in i to be at most the probability that a helicopter from the closest base reaches i on time. Note that actually, equality should hold in (4), and equality will in fact be obtained in the optimal solution (due to the nature of our objective).

The model above was used to perform greenfield analysis. For brownfield analysis, we added a constraint that the sum of x_j for j in the set of existing base locations is greater or equal to some number. That means at least a certain number of bases must end up at existing base locations.

To solve the model with Gurobi, we used a linear approximation that is described in (14). This approach is summarized as follows. First observe that each of the summed terms in the objective (u_i^{1-a}) is concave for any a between 0 and 1. This concavity allows to construct a set of upper bounds by creating linear functions that are that are tangential to u_i^{1-a}. We started by creating five such linear upper bounds per municipality, at values logarithmically spaced between 0 and 1. These five lines together create a piecewise linear approximation of the true shape of u_i^{1-a}. We then let Gurobi solve the model with this linear objective. We then evaluate the solution: if for any municipality the value attained in the linear approximation differs more than ε from the value in the true objective function, we add another linear upper bound tangential to u_i^{1-a} at the value u_l attained in the solution. We iterate this process until for each location l the value u_i^{1-a} is approximated with an error of at most ε. In our implementation we used ε = 0. 00001.
