# Supplementary material for: Introducing fairness in Norwegian air ambulance base location planning
Source: Scand J Trauma Resusc Emerg Med. 2021 Mar 20;29:50. doi: 10.1186/s13049-021-00842-0 (PMC7980553; doi:10.1186/s13049-021-00842-0)
Supplement: Supplementary file 2 — Additional file 2. [file 13049_2021_842_MOESM2_ESM.pdf]

Proof of convergence: iso-elastic SWF $\rightarrow$ Bernoulli-Nash SWF

Throughout this document we use the following notation:
d_i is the fraction of patients (demand) in location i.
u_i is the utility in location i.
f_{iso} is the iso-elastic social welfare function.
f_{BN} is the Bernoulli-Nash social welfare function.

The iso-elastic Social Welfare Function (SWF) in our paper is denoted

$$\left(\frac{1}{1-a}\right)\sum_{i=1}^{N} d_i u_i^{1-a} \tag{1}$$

where a is a constant, $a \geq 0, a \neq 1$.

At this stage it is worthwhile to note that there exists an alternative definition in literature [1]. We next introduce this, as this allows us to show convergence:

$$\left(\sum_{i=1}^{N} d_i u_i^{1-a}\right)^{\frac{1}{1-a}}, \tag{2}$$

We point out that, although the two formulas above are different, optimal solutions are the same (for any $0 \leq a \leq 1$). This can be seen by observing that for both formulas the optimum is found by maximizing $\sum_{i=1}^{N} d_i u_i^{1-a}$.

[1] contains a brief statement that (2) converges to the Bernoulli-Nash SWF as $a \rightarrow 1$. We next create a proof with more details.

The approach is to show that $\log(f_{iso})$ converges to $\log(f_{BN})$, as follows:

$$\begin{aligned}\lim_{a\to 1}\log(f_{iso}) &= \lim_{a\to 1}\log\left(\left(\sum_{i=1}^{N} d_i u_i^{1-a}\right)^{\frac{1}{1-a}}\right)\\ &= \lim_{a\to 1}\frac{\log\left(\sum_{i=1}^{N} d_i u_i^{1-a}\right)}{1-a}\end{aligned}$$

which we will write as: $\lim_{a\to 1}\frac{f(a)}{g(a)}$,

where $\lim_{a\to 1} f(a) = 0$ and $\lim_{a\to 1} g(a) = 0$, so applying l'Hopital's rule gives:

$$\begin{aligned}
\lim_{a\to 1}\frac{f(a)}{g(a)} &= \lim_{a\to 1}\frac{f'(a)}{g'(a)} \\
&= \lim_{a\to 1}\frac{\sum_{i=1}^{N} d_i u_i^{1-a}\log(u_i)}{\sum_{i=1}^{N} d_i u_i^{1-a}} \\
&= \sum_{i=1}^{N} d_i \log(u_i) \\
&= \log(\prod_{i=1}^{N} u_i^{d_i}) \\
&= \log(f_{BN}).
\end{aligned}$$

References

[1] Oded Stark, Fryderyk Falniowski, and Marcin Jakubek. Consensus income distribution. *Review of Income and Wealth*, 63(4):899–911, 2017.
