# Supplementary material for: Introducing fairness in Norwegian air ambulance base location planning
Source: Scand J Trauma Resusc Emerg Med. 2021 Mar 20;29:50. doi: 10.1186/s13049-021-00842-0 (PMC7980553; doi:10.1186/s13049-021-00842-0)
Supplement: Supplementary file 3 — Additional file 3. [file 13049_2021_842_MOESM3_ESM.pdf]

Additional computations, supplemental to:
C. J. Jagtenberg et al., Introducing fairness in Norwegian air ambulance base location planning, SJTREM, doi: 10.1186/s13049-021-00842-0

In this file we further elaborate on which experiments were performed. Note that the true distribution of flight times for Norwegian HEMS are unknown. Initially we ran the optimization algorithm for a range of different models and statistical distributions of flight time:

- Uniformly distributed flight times, with up to 20% and up to 30% deviation from the expected value.
- Normally distributed flight times, with a coefficient of variation of 0.1, 0.2 and 0.3.

When deciding between all of these options, the authors' intuition - based on hands-on experiences with working in other industries - was that in a uniform distribution 30% variation would be rather much, and the true value would more likely be closer to 10%. The normal distribution with coefficient of variation 0.1 turned out to give very similar results to the uniform distribution with up to 10% deviation. (For larger variations, the uniform and the normal distribution started to show their differences more.) As a previous reference hinted that a uniform distribution might be more realistic, we chose to let the paper show results for that one. In summary, the chosen distribution is plausible, is related to existing literature, and gives similar results as other plausible models.
